# Improvement of Compressibility and Thaw-Settlement Properties of Warm and Ice-Rich Frozen Soil with Cement and Additives

**DOI:** 10.3390/ma12071068

**Published:** 2019-04-01

**Authors:** Mingtang Chai, Jianming Zhang

**Affiliations:** State Key Laboratory of Frozen Soil Engineering, Northwest Institute of Eco-Environment and Resources, Chinese Academy of Sciences, Lanzhou 730000, China; cmt620422@163.com

**Keywords:** permafrost, compression coefficient, scanning electron microscope (SEM), thaw strain, calcium silicate hydrate

## Abstract

The warm and ice-rich frozen soil (WIRFS) that underlies roadway embankments in permafrost regions exhibit large compression and thaw deformation, which can trigger a series of distresses. Cement and additives were used in this study to improve the compressibility and thaw-settlement properties of WIRFS. We, therefore, selected optimum additives and studied the improvement effect on the frozen soil with 30% water content based on our previous research. Given constant load and variable temperatures, compression coefficients, thaw strains, and water content changes were obtained at temperatures of −1.0 °C, −0.5 °C, and 2.0 °C to evaluate the effect of improvements. A scanning electron microscope (SEM) was then used to observe the microstructure of improved soils and analyze causal mechanisms. Data show that hydration reactions, physical absorptions, cement, and additives formed new structures and changed the phase of water in frozen soil after curing at −1.0 °C for 28 days. This new structure, cemented with soil particles, unfrozen water, and ice, filled in the voids of frozen soil and effectively decreased the WIRFS compression coefficient and thaw strain.

## 1. Introduction

Warm and ice-rich frozen soil (WIRFS) is a kind of substrate that has a temperature above −1.5 °C and an ice content above 20% [1], as well as high environmental sensibility, low mechanical strength, large compressibility, and huge thaw settlement [2,3]. Because of the existence of the WIRFS, permafrost regions have enhanced the occurrence of geological hazards and asymmetrical embankment settlements of roadways [4,5]. These phenomena have affected operational safety and increased the maintenance cost of roadways [6]. Studies on the compressibility and thaw settlement of WIRFS is therefore significant to calculate embankment settlement and analyze deformation characteristics.

Laboratory experiments and field measurements, as well as research on the compressibility and thaw settlement of WIRFS, show that both compression strain and the compression coefficient are proportional to ice content, temperature, and load. The compression coefficient is also known to increase exponentially in concert with temperature [3,7]. Thus, ice-rich frozen soil settlement during thawing is relative to dry density and the compression coefficient, based on the calculation model of the total settlement of the embankment that can be constructed [8]. The embankment settlement of WIRFS is therefore exponential to the thickness of these features as well as the thickness of the ice-rich permafrost layer and the rate of temperature increase. We also know that settlement deformation comprises three stages that are interactive and simultaneous, thawing settlement, creep, and settlement caused by freezing and thawing [9,10].

To solve the problems listed above, thermosyphons, ventilated ducts, and crushed rock (“cooling measures”) have been adopted in the construction of embankments in permafrost regions. Long-term measurement data have also shown that these measures effectively decrease the temperature of permafrost as well as embankment settlements by regulating solar radiation, thermal convection, and conduction [11,12,13,14]. In addition to the physical measures outlined above, a number of chemical methods to improve the physical and mechanical properties of soils have been proposed on the use of hydraulic materials and additives. Soil stabilization is generally a technique in which cementing agents (e.g., P·O 42.5, lime, asphalt, and other solid industrial by-products) are added to the soil to improve their physical and mechanical properties. These materials can be divided generally into six classes, such as industrial wastes, nano industrial wastes, agriculture-farming wastes, aquaculture-farming wastes, natural minerals, dust and powders [15]. The replacement of clinker in cement blends not only reduces the consumption of resources and energy but also avoids the environmental burden associated with clinker production [16].

As a result of the lower cost and better performance, stabilized soils generate well-controlled and superior properties via the addition of selected materials [17]. Chemical improvements have commonly been adopted in the case of seasonally frozen soils to decrease strength loss after the freeze-thaw cycle [18,19,20,21]. Two commonly used construction techniques have been applied in this context including chemical improvements, the pre-treatment of natural permafrost before construction, and improvements to subgrade frozen soils beneath existing constructions. The application of chemical improvements are rare in permafrost regions; however, due to ice, unfrozen water, and negative frozen soil temperatures, the materials and additives selected for improvements should not only prevent the freezing point of water being depressed but should also be active at negative temperatures. Selecting optimal materials and additives is therefore key to improve the compressibility and thaw settlement of WIRFS. We, therefore, compared the effects of nine additives on the unconfined compressive strength of the WIRFS based on our previous research and obtained a range of appropriate additives that are effective for improvements [22]. We prefer the second method above as this means that the chemical improved frozen material were served as subgrade soils for applications in permafrost regions.

Effective additives in cement were selected in this paper based on our previous research. We, therefore, analyzed the effects of improving the cement and additives on the compressibility and thaw-settlement properties of frozen soil with 30% water content under constant load and variable temperatures. We, therefore, obtained compression coefficients, thaw strains, and the change characteristics of water content to evaluate improvement effects. Hydration products and the microstructure of improved soils were observed using scanning electron microscopy (SEM) in this study to explain improvement mechanisms.

## 2. Materials and Methods

### 2.1. Experimental Materials

**Figure 1:**
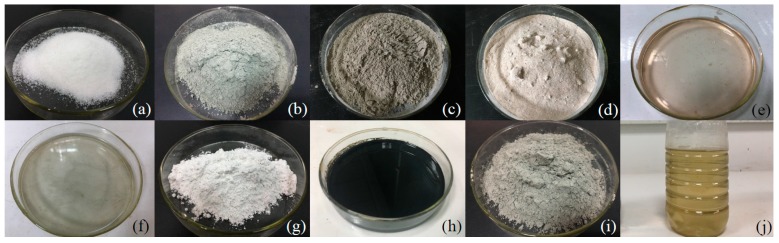
Cements and additives, (**a**) super absorbent polymer (SAP), (**b**) ordinary Portland cement (PO), (**c**) fly ash, (**d**) activator of the geopolymer, (**e**) anti-freezing agent (AFA), (**f**) early strength agent (ESA), (**g**) metakaolin (MK), (**h**) EN-1, (**i**) inorganic additive from Xi’an, (**j**) Toogood.

Silty clay samples from the Beiluhe Basin on the Qinghai-Tibet Plateau adjacent to the Qinghai-Tibet Highway and Railway (Qinghai-Tibet Plateau, China) were selected and crushed after air-drying and filtered through a 2 mm sieve. Particle size distributions within samples, as well as their physical and chemical properties, are shown in Table 1 and additives selected for evaluation in this study were super absorbent polymer (SAP), ordinary Portland cement (P·O 42.5), geopolymer (GP), anti-freezing agent (AFA), early strength agent (ESA), metakaolin (MK), and three soil stabilizers (i.e., EN-1, an inorganic additive (IA), and Toogood). The phase composition of used cement is C3S, C2S, C3A, and C4AF. The chemical composition of used cement is shown in Table 2. The main components of the SAP (Figure 1a) were sodium polyacrylate (77.0% by weight) and polyacrylic acid (23.0% by weight); thus, one gram of SAP can absorb 400 g distilled water or 40 g 0.9% NaCl solution. In contrast, P·O 42.5 is shown in Figure 1b, while GP is composed of 59.4% fly ash (by weight, Figure 1c) from Yulin, China, 4.9% alkali activator (by weight, Figure 1d) and 35.7% ordinary P·O 42.5 (by weight). The class of used fly ash is F, and the physical properties and chemical composition of fly ash and MK are shown in Table 3 and Table 4. The alkaline activator will provide an environment of this type under high concentrations and will also dissolve the Si-Al gel, while at the same time will also act to balance redundant negative charge. The AFA (Figure 1e) and ESA (Figure 1f) used in this study are both commercial products, while the MK (Figure 1g) was from Xingtang, China. In contrast, EN-1 (Figure 1h) is a hybrid polymer imported from America that contains 6.0% surfactant and has a pH of 0.7, while the IA we used was sourced from the Xi’an Highway Research Institute (Xi’an, China) and was mixed with P·O 42.5 at the dosage of 2%. The component of IA is NaOH and Na_2_SiO_3_ with the ratio of 1:0.43 by weight. The mixture of this component and cement is shown in Figure 1i. The Toogood additive, in this case, was produced by a Chinese company in Hunan Province (Figure 1j); this compound is an ionic soil stabilizer that has a pH of 11.13.

The dosage of each additive was determined based on our previous research [22] (Table 5). Thus, to analyze the improvement effects of cement on frozen soil and to compare these with other additives, three soil samples with 5%, 15%, and 30% cement but no additives were compared in this study. The GP contains cement, fly ash, and an alkali activator and was applied at a 15% dosage, while SAP was used at the same concentration and the rest were used at a 15% content. The dosages of SAP, cement, and GP were applied via soil dry weight, while the remainder of additives concentration was determined via cement dry weight.

### 2.2. Experimental Method

Soil samples were prepared in a cold room at approximately −5.0 °C, although there were some differences throughout this process in the preparation and subsequent supplement of powder and liquid chemical additives. Ice was then crushed and filtered through a 2 mm sieve before being mixed with soil, cement, and powder additives. The ratio between soil and ice, in this case, was 1:0.3 by mass and enabled an initial water content of 30%. In the case of liquid chemical additives, the ratio of soil, ice, and water was 1:0.2:0.1; thus, liquid chemical additives were diluted with distilled water and uniformly mixed with soil, ice, and cement. These mixtures were then homogeneously compacted and molded into cylindrical samples at two sizes (i.e., 61.8 mm diameter and 40 mm height alongside 61.8 mm diameter and 100 mm height), which had a dry density of 1.25 g/cm^3^. Small soil samples were then used for compression tests while large soil samples were used for the measurement of unfrozen water content. Samples were wrapped tightly with plastic film and cured in an incubator at −1.0 °C for 28 days. Finally, to prevent soil moisture from evaporating, the time used for sample preparation was less than 30 min. Three identical samples for each additive and dosage level were prepared to ensure the reliability of test results.

**Figure 2:**
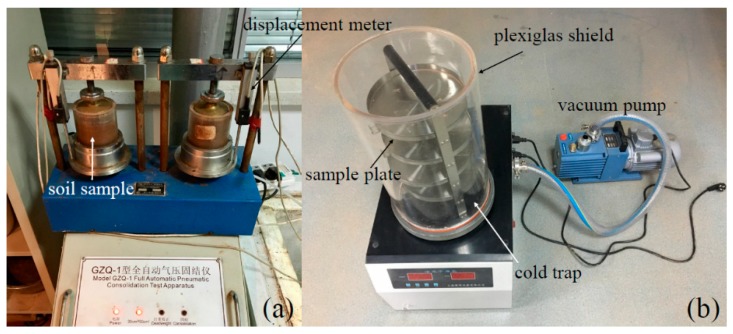
The consolidation apparatus (**a**) and vacuum freeze dryer (**b**) used in this study.

Compression tests were conducted using a Model GZQ-1 Full Automatic Pneumatic Consolidation Test Apparatus (Shanghai Testing Instrument Company, Shanghai, China, Figure 2a) under constant loads and variable temperatures following the Standard for Soil Method (GB/T 50123-1999). Filter papers and porous stones were placed at both ends of clays and were placed into consolidation apparatus alongside samples. Before compression tests, soil samples were placed at −1.0 °C for curing; during these tests, samples were placed in a consolidation test apparatus and were maintained at a constant testing temperature within an incubator. Experimental loads were set at 0.1 MPa, 0.2 MPa, and 0.3 MPa, while temperatures were set at −1.0 °C, −0.5 °C, and 2.0 °C, respectively. Thus, when deformation was less than 0.025 mm per hour, the temperature was turned into the next level and testing data were automatically collected using a computer. A field emission SEM was then used to observe the form of hydration products and soil microstructure. In order to eliminate the change in soil structure during the air-drying process, a vacuum freeze dryer (FD-1A-50, produced by Shanghai Yuming Biotechnology Co., Ltd., Shanghai, China, Figure 2b) was used. After thaw compression, all samples prepared for SEM were freeze-dried via vacuum sublimation. The type of SEM is SU8020 (Hitachi, Tokyo, Japan), which is the field emission scanning electron microscope with high resolution.

Changes in the soil water content were finally measured via a four-part process that included the change in total soil sample water content after 28 days curing, the content of unfrozen water at three testing temperatures, the change in water content before, and after, thaw compression, and the change in water content during drying in an oven after this process was complete. The change of total water content after curing was then measured by oven drying at 108 °C for 24 h, and unfrozen water content at −1.0 °C, −0.5 °C, and 2.0 °C were measured using time domain reflectometry. A soil moisture sensor (CSF11, produced by Beijing Star Sensor Technology Co., Ltd., Beijing, China) was inserted into the middle of each soil sample; before testing, samples were frozen completely at −20 °C. All samples were placed into an incubator for 6 h to make the temperature of samples constant and uniform. Thus, as the temperature of soil samples were held constant and equal to testing temperature, the probe signal remained steady, but after thaw compression, samples were dried in an oven at 108 °C for 24 h to measure the change of water content during compression. After freeze drying, samples were then dried again in an oven at 108 °C for 24 h to measure the decomposition volume of crystal water in hydration products.

## 3. Results

### 3.1. Compression Strain Characteristics

**Figure 3:**
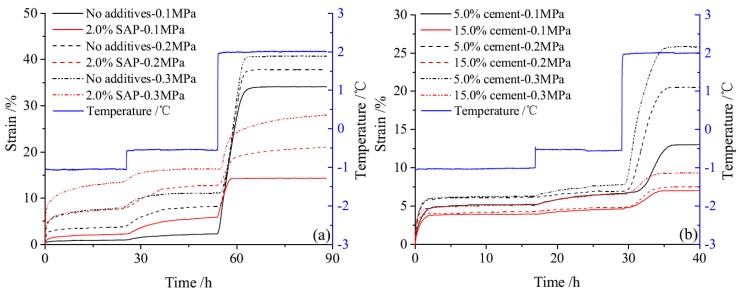

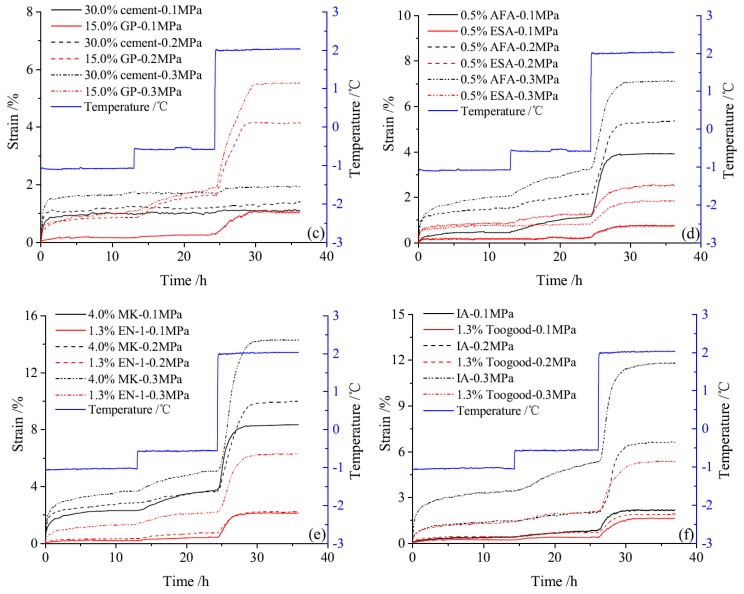
Compression strain curves under constant loads and at different temperatures (i.e., (**a**): soil samples without additives and with 2.0% SAP; (**b**): soil samples with 5.0% cement and 15.0% cement; (**c**): soil samples with 30.0% cement and 15.0% GP; (**d**): soil samples with 0.5% AFA + 15.0% cement and 0.5% ESA + 15.0% cement; (**e**): soil samples with 4.0% MK + 15.0% cement and 1.3% EN-1 + 15.0% cement; (**f**): soil samples with IA + 15.0% cement and 1.3% Toogood + 15.0% cement).

The thaw compression curves of soil samples with different additives under each load and at each temperature are shown in a sequence in Figure 3. Data show that, on the whole, except SAP, cement and additives shortened the lasting deformation time in each case. The total strain under three loads increase in concert with temperature, and data show that characteristics at negative temperatures are similar. Results also show that strain at −1.0 °C is smaller than that at −0.5 °C, but then increases rapidly at 2.0 °C; in contrast, the strain rate of soil samples with cement and additives is significantly smaller in cases without cement or additives during thawing.

**Figure 4:**
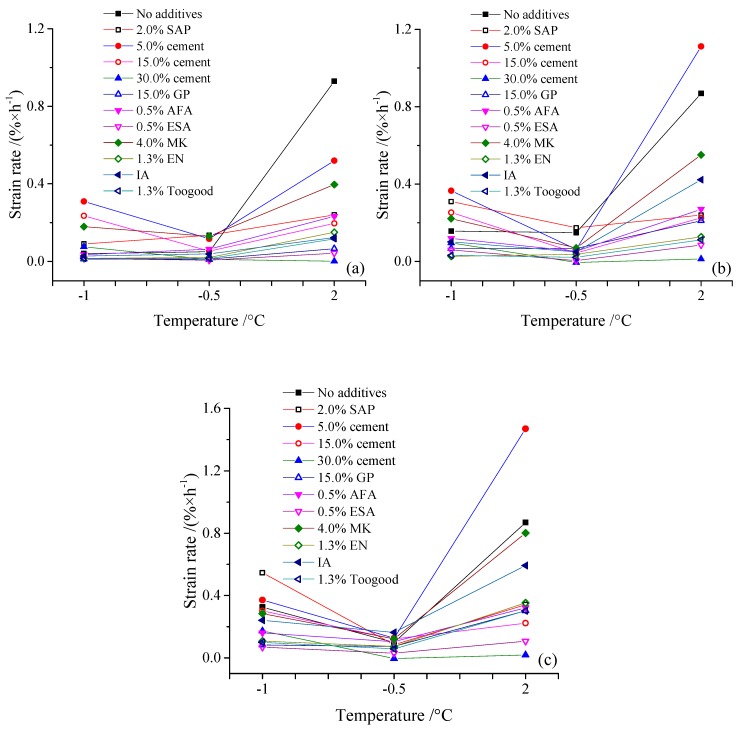
Soil sample strain rates at −1.0 °C, −0.5 °C, and 2.0 °C under 0.1MPa (**a**), 0.2MPa (**b**), and 0.3 MPa (**c**).

The characteristics of strain change before, and after, thawing during compression tests are not the same. Results show that before thawing, at temperatures of −1.0 °C and −0.5 °C, strain increases in concert with load, while compared to samples that lack cement and additives, SAP injection increased strain, while cement and geopolymer decreased this variable in samples. Data also show that strain decreases as cement dosage increases, while the additives, including ESA, EN-1, and Toogood, within the material, improved its performance and decreased the strain to a greater extent. All of these additives, therefore, exerted the best effects to improve soil samples. At the same time, after thawing at 2.0 °C and compared with unimproved samples, cement, and all additives effectively decreased thaw strain. The characteristics of thaw strain between samples with 15.0%, 30.0%, and 15.0% cement plus 0.5% ESA were almost the same. Thus, among the additives in cement, ESA performed best, followed by MK and IA; the strain rate of soil samples at −1.0 °C, −0.5 °C, and 2.0 °C under 0.1 MPa, 0.2 MPa, and 0.3 MPa are shown in Figure 4. As shown in these figures, at −1.0 °C and −0.5 °C, most additives decreased the strain rate of soil samples under the three loads, while at 2.0 °C, other additives effectively decreased the strain rate, with the exception of samples with 5.0% cement under 0.2 MPa and 0.3 MPa.

### 3.2. Water Content Change Characteristics

**Figure 5:**
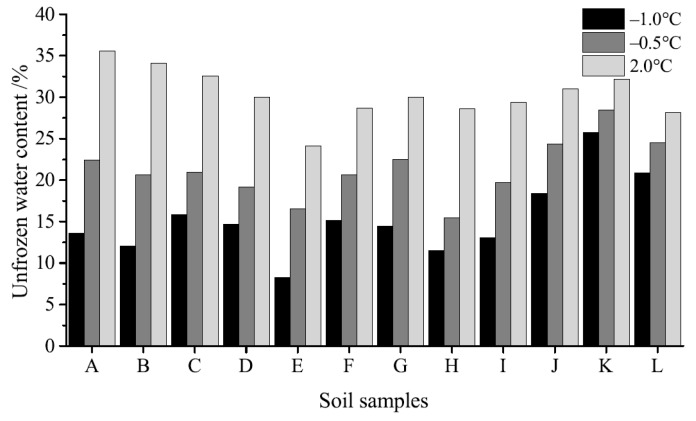
Unfrozen water content at −1.0 °C, −0.5 °C, and 2.0 °C: (A) No additives; (B) 2.0% SAP; (C) 5.0% cement; (D) 15.0% cement; (E) 30.0% cement; (F) 15.0% GP; (G) 15.0% cement + 0.5% AFA; (H) 15.0% cement + 0.5% ESA; (I) 15.0% cement + 4.0% MK; (J) 15.0% cement + 1.3% EN-1; (K) 15.0% cement + IA; (L) 15.0% cement + 1.3% Toogood.

The unfrozen water contents of soil samples at −1.0 °C, −0.5 °C, and 2.0 °C are shown in Figure 5; these data show that this variable increases in concert with temperature. Therefore, except SAP, the results show that the hydration of cement and additives consumed the unfrozen water in samples and reduced the total water content at 2.0 °C. These additives also depressed the freezing point of water in samples and resulted in an increase in unfrozen water content at low cement dosages (5.0% and 15.0%). Data show that at high cement dosages (30%), both total water content and unfrozen water content decreased; comparisons with soil samples without cement and additives show that the addition of SAP lowered both unfrozen and total water content. Indeed, when the cement dosage was 15%, EN-1, IA, and Toogood increased the unfrozen water content of soil samples to the largest extent.

**Figure 6:**
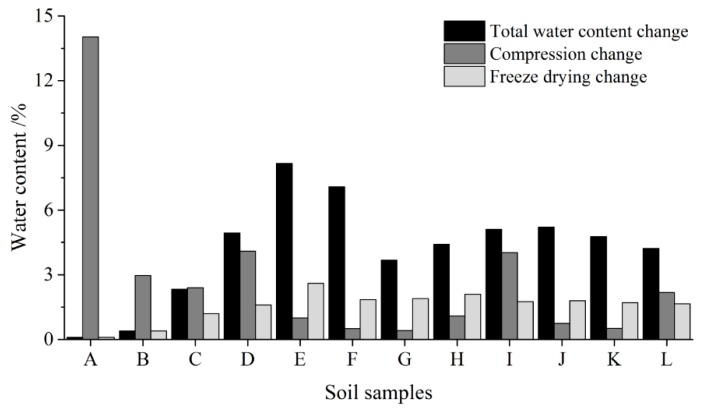
Changes in water content (i.e., changes in total water content after curing, changes in total water content after thaw compression as well as changes in crystal water content after freeze-drying and the number of soil samples (the number in axis is same as Figure 5)).

The characteristics of water changes in soil samples during, and after, compression are shown in Figure 6. Thus, comparing samples that lack both cement and additive, the addition of both of these factors effectively decreases the water drainage of soil samples during thaw and compression. The water in the samples was therefore solidified by hydration products in these cases. Similarly, by comparing the 15% cement samples, it is clear that MK performs poorly in terms of water decrease during thaw compression. The change in sample water content after thaw compression was larger than in other samples in this case. Indeed, except SAP, cement and the other additives also decrease the total water content of samples after curing. During the oven drying process, data reveal small changes in water content via evaporation due to the decomposition of unstable crystal water in hydration products. Differences in water changes during drying between samples are not obvious, however.

### 3.3. Sample Microstructural Changes Following Improvements

**Figure 7:**
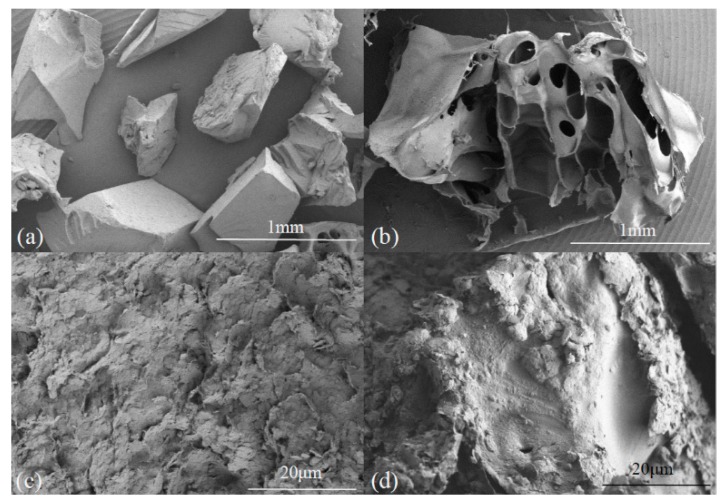
SEM pictures after thaw compression: (**a**) SAP particles before absorption; (**b**) SAP particles after absorption; (**c**) a soil sample without cement or additives; (**d**) a soil sample with 2.0% SAP.

The main SAP component identified in this paper is sodium polyacrylate. Data show that given the dissociation of –COONa^+^, macromolecular chains expanded and swelled in the presence of water. The difference of ionic concentration inside, and outside, of these chains means that SAP performs as a hydrogel under osmotic pressure. The swollen gel strength, in this case, can be high enough to retain a large amount of water [23]; SEM pictures of SAP show that that irregular and angular particles (Figure 7a) become fluffy (Figure 7b) after absorption in this case. These pores can absorb and retain plenty of water, while at the same time, the structure of this additive after absorption will cause a lower change in water content during thaw and compression under the external load.

SEM pictures of soil samples without cement or additives are shown in Figure 7c after thaw and compression. These soil particles are dense and bond with each other closely, leaving few voids, while the compression coefficient and thaw strain of samples are larger than their counterparts with cement and additives. The soil particles of samples with SAP are not so dense because of the filling effect of hydrogels from this additive (Figure 7d). The skeleton brace of SAP after the absorption lowered the thaw strain of the sample.

**Figure 8:**
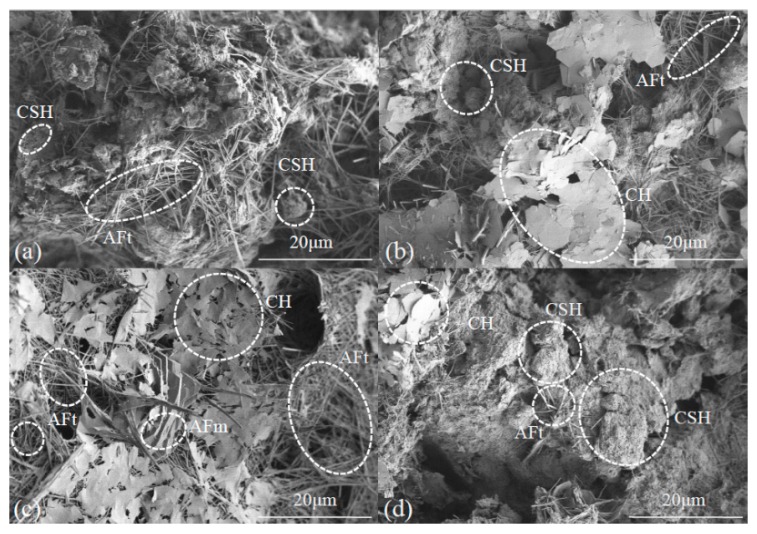
SEM pictures of soil samples following thaw compression: (**a**) 15.0% cement; (**b**) 15.0% cement + 0.5% AFA; (**c**) 15.0% cement + 0.5% ESA; (**d**) 15.0% cement + 4.0% MK.

The SAP additive decreases thaw strain via physical absorption, while cement and other additives decreased the strain by hydration reaction. The SEM picture of soil samples with cement (Figure 8a) show the flocculated calcium silicate hydrates (CSH) and the needle-shaped ettringite (AFt) displayed in the cement-stabilized soil matrix. Some of the CSH attached to the surface of soil particles comprise filled-in voids and soil particles bonded with one another. The AFt components are irregularly distributed and braced among soil particles, while cement stabilized soils contain more pores and have a lower compression strain than others without cement or additives. The addition of AFA also generated a larger amount of hydration products via a more complete reaction than cement stabilized soil. Flake-shaped calcium hydroxide (CH) is also present while AFt also performed more densely in samples (Figure 8b).

An SEM image of a soil sample with cement and ESA is shown in Figure 8c; this image shows that, compared with samples containing cement, the addition of ESA generated more CH, which intersected with denser Aft, while monosulfate (AFm) also appeared in the sample, converted from the ettringite phase and it is the final production of hydration of PO 42.5 [24]. Data show that ESA improved hydration rate, promoted the hydration process to the next stage, and lowered the thaw compression strain of soil sample. At the same time, the addition of MK increased the amount of CSH and aggregated soil particles (Figure 8d).

**Figure 9:**
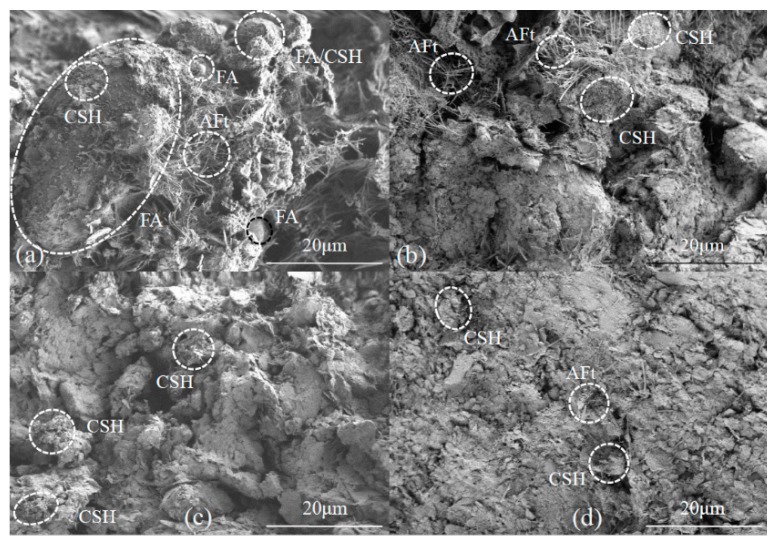
SEM pictures of soil samples following thaw compression: (**a**) 15.0% GP; (**b**) 15.0% cement + 1.3% EN-1; (**c**) 15.0% cement + IA; (**d**) 15.0% cement + 1.3% Toogood.

Results show that both AFt and CSH occur in the soil and GP mix (Figure 9a), while unreacted and partially reacted fly ash particles were also present in the sample after curing at −1.0 °C for 28 days. We also show that unreacted fly ash particles perform as solid spheres because their diameters are distributed from several micrometers to hundreds of micrometers, while CSH gels are also cemented onto the surface of partially reacted fly ash particles [25]. An SEM picture of samples with cement and EN-1 (Figure 9b) show that this entity is similar to the sample with cement and MK, while CSH and AFt are displayed in the aggregation surface of soil particles. Contrasting SEM pictures of soil samples with IA and Toogood are shown in Figure 9c,d; the stimulation and promotion of activators in additives resulted in more hydration products. This means that the cementation of hydration gels with soil particles comprise a hardened skeleton to decrease compression and thaw-settlement of samples.

## 4. Discussion

### 4.1. Compression Coefficient

The compression coefficient was used in this paper as a proxy for stabilized soil characteristics. Applying the Standard for Soil Test Method (GB/T50123-1999), a compression coefficient under load between 0.1 MPa and 0.2 MPa was used as the standard in this analysis; this is based on the compression coefficient, *m_v_*, as follows:

*m_v_* = *Δh*/*h* × *ΔP* (1)

In this equation, *m_v_* denotes the compression coefficient (MPa^−1^), *Δh* is the total deformation of soil sample under loads between 0.1 MPa and 0.2 MPa (mm), *h* is the initial height of sample (mm), and *ΔP* is the difference between the two loads (MPa).

**Figure 10:**
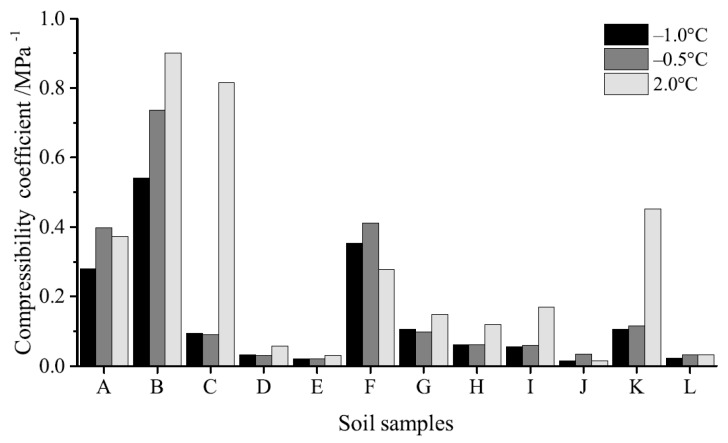
Sample compression coefficients (the soil sample number order is the same as Figure 5).

The compression coefficients of soil samples under loads of 0.1 MPa, 0.2 MPa, and 0.3 MPa are shown in Figure 10. These data show that the compression coefficient of samples containing 5.0% cement and 15.0% cement + IA are larger than those for the sample without cement and additive at 2.0 °C, while both cement and additives decreased this coefficient at three testing temperatures in the remainder of cases. Indeed, compared with samples containing 15.0% cement, just the compression coefficients of those that comprises 15.0% cement + 1.3% EN-1 and 15.0% cement + 1.3% Toogood were lower than this value overall. This means that the change characteristics of compressibility can be divided into three categories when all samples are compared, the compression coefficient at a positive temperature is higher than at a negative temperature (i.e., samples containing 5.0% cement, 15.0% cement + 0.5% AFA, 15.0% cement + 0.5% ESA, 15.0% cement + 4.0%, MK, and 15.0% cement + IA), while the compression coefficient at a positive temperature is lower than at a negative temperature (i.e., the sample containing 15.0% GP). Similarly, the compression coefficient at a positive temperature is almost equal to that seen at a negative temperature (i.e., samples containing 15.0% cement, 30.0% cement, 15.0% cement + 1.3% EN-1, and 15.0% cement + 1.3% Toogood).

### 4.2. Modification Mechanism

Additive components can be divided into four categories, organic (SAP), inorganic (MK and IA from Xi’an, China), ionic (EN-1 and Toogood), and composites (AFA and ESA). The mechanisms that underlie their modification can be divided into physical (SAP) and chemical modifications, while the remainder are additives (even though the effects of these additives are different in each case). In soil samples that lack either cement or additives, particles, the ice skeleton and unfrozen water bonded with one another and formed structures with a certain strength. We show that the addition of cement and additives changes the original structure of frozen soils; SAP particles and hydration products distributed and filled in voids within samples to form new structures. These additions also changed soil sample compression and thaw-settlement properties.

After absorption, SAP strength was also lower than both the soil and ice skeleton at negative temperatures; this means that the compression coefficient of samples treated with SAP is larger those either without cement or additives. Thaw strain was also decreased by SAP water retention and so the addition of cement to soils has three effects: (1) the cement reacts with unfrozen water within soils at −1 °C such that hydration products wrapped and filled particle pores, and the bracing effect of products decreased compression strain; (2) Ca^2+^, Na^+^, and K^+^ cation exchange dissociated from calcium hydroxide in cements and soils to thin the electrical surface double layer and lower particle dispersity leading to higher aggregation, and; (3) Ca(OH)_2_ from the hydration of cement participated in a pozzolanic reaction with SiO_2_ and Al_2_O_3_ from clay minerals and generated CSH and hydrated calcium aluminate [26]. The main hydration processes in soil samples, therefore, include the fact that tricalcium silicate (C_3_S) and dicalcium silicate (C_2_S) in cement will generate tobermorite (CSH) with water (Equations (2) and (3)), while C_3_S and gypsum generate ettringite (AFt) (Equation (4)) [27]. In cases where the gypsum is completely consumed, AFt will transform into AFm (Equation (5)) [28], as follows:

2Ca_3_SiO_5_ + 6H_2_O = Ca_3_Si_2_O_7_·3H_2_O + 3Ca(OH)_2_ (2)

2Ca_2_SiO_4_ + 4H_2_O = Ca_3_Si_2_O_9_·3H_2_O + Ca(OH)_2_ (3)

Ca_3_Al_2_O_6_ + 3CaSO_4_·2H_2_O + 26H_2_O = Ca_6_Al_2_(SO_4_)_3_(OH)_12_·26H_2_O (4)

Ca_6_Al_2_(SO_4_)_3_(OH)_12_·26H_2_O + 2Ca_3_Al_2_O_6_ + 10H_2_O = 3Ca_4_Al_2_(SO_4_)·14H_2_O (5)

After the addition of GP, the presence of an alkali-activator reveals that a new three-dimensional inorganic amorphous structures of Si–O tetrahedron and Al–O octahedron is produced by the breaking of original Si–O and Al–O bonds in fly ash and cement samples. The fly ash, in this case, enabled the presence of additional nucleation sites for cement hydration to increase the performance of stabilized frozen soil [29]. In this context, as AFA, ESA, EN-1, IA, and Toogood are all commercial products, their chemical components are well known, and so each additive effectively promoted these processes and the amount of cement hydration observed in SEM pictures. The improvement mechanisms caused by MK can be explained by the fact that this additive increased the amount of CSH, decreased the amount of CH, and the ratio of Ca/Si in the matrix [30,31].

Throughout the curing of frozen soil samples, complicated phase changes in water occurred in the hydration of cement. The absorption of SAP is a physical process and is reversible; total water content, therefore, remains constant after curing. On the one hand, cement and additives depressed the freezing point of water and increased the content of unfrozen water at the same temperature, while liquid water at the same time was transformed to crystal form after hydration, as this had a bracing effect with hydration products. The total water content decreased after the curing. In the thaw compression, some liquid water drained out. We show clearly that freeze-drying occurred while the ice was sublimated; this means that only crystal water was retained in the hydration products as most of this decomposes at extremely high temperatures and little evaporates at 108 °C [32].

## 5. Conclusions

The data presented in this study show that the addition of SAP at 0.1 MPa doubled both compression strain and compression coefficient, but also decreased the thaw strain between 34% and 14%. The remainder of the additives discussed here also improved cement hydration processes and decreased the compressive strain and thaw-settlement of soil samples. We show that when cement dosage is 15%, ESA, EN-1, and Toogood exhibit the best improvement effects across our samples. The addition of these three additives in cement decreased the compression coefficient from about 0.35 MPa^−1^ on average to about 0.03 MPa^−1^ on average and decreased the thaw strain from 34% to 2% at 0.1 MPa.

Supplements with cement and additives all changed the water phase in frozen soils via hydration reactions. Additives, therefore, decreased the total water content after curing (except for SAP) as well as after thaw compression. The crystal water, along with the hydration products, formed a new matrix in the frozen soils, while GP, EN-1, and IA have the best effect on water content decreases, in most cases up to a 5% maximum.

SEM pictures show that large amounts of AFt are present in hydration products after the addition of cement and additives. The volume of AFt expands after the formation to the extent that one of these molecules encompasses 32 water molecules. AFt decreased total water content after curing and increasing the strength of soil samples. The amount of CH increases within samples treated with AFA; thus, AFm appeared in samples with ESA, which indicates that the hydration process was conducted to the next stage. Other additives in cement have increased the amount and density of CSH and promoted the rate and process of hydration reaction. The hydration products of cement and additives are not uniformly distributed in the stabilized soils; particles were flocculated and agglomerated by the cation-exchange in these experiments, while hydration products cemented and filled in voids.

## Figures and Tables

**Table 1:** Particle-size distributions as well as the physical and chemical properties of soil samples.

Soil Type	Particle-Size Distribution/%				Plastic Limit	Liquid Limit	pH	Major Ions
Siltyclay	>0.1 mm	0.1~0.05 mm	0.05~0.005 mm	<0.005 mm	18.6	36.7	8.59	Na^+^, SO_4_^2−^
	3.69	11.96	52.83	31.52				

Note: % means by weight.

**Table 2:** The chemical composition of used cement (%).

CaO	SiO_2_	Al_2_O_3_	Fe_2_O_3_	MgO	SO_3_	R_2_O
62.14	21.24	5.42	3.72	1.74	2.57	0.57

Note: R_2_O means monovalent oxide.

**Table 3:** The physical properties of metakaolin and fly ash.

Physical Properties	Bulk Density (mg/kg)	Dry Density (g/cm^3^)	Total Porosity (%)	Specific Surface Area (m^2^/g)
Metkaolin	0.75	2.61	51.21	3.41
Fly ash	0.60	2.08	71.12	2.12

**Table 4:** The chemical composition of used metakaolin and fly ash (%).

Component	SiO_2_	Al_2_O_3_	CaO	K_2_O	Fe_2_O_3_	Na_2_O	MgO	Others
Metakaolin	41.00	43.50	0.40	3.20	0.40	0.40	0.30	10.80
Fly ash	44.56	28.45	7.98	1.45	5.21	0.52	0.85	10.98

**Table 5:** Cement and additive dosages.

Item	Cement	GP	Additives					
			SAP	AFA	ESA	MK	EN-1	Toogood
Dosage/%	5.0 15.0 30.0	15.0	2.0	0.5	0.5	4.0	1.3	1.3
